# Long-Term Outcome of Intraoperative Radiotherapy for Early-Stage Breast Cancer

**DOI:** 10.3390/cancers18040699

**Published:** 2026-02-20

**Authors:** Eyal Bratt, Orit Pasternak, Daphne Levin, Yonina Tova, Vladislav Grinberg, Moshe Papa, Mordechay Gutman, Svetlana Zalmanov, Raphael Moshe Pfeffer, Roxolyana Abdah-Bortnyak, Merav Akiva Ben-David

**Affiliations:** 1 Department of Oncology, Assuta Medical Center, Tel-Aviv 6971028, Israel; eyal.bratt@campus.technion.ac.il (E.B.); meravak@assuta.co.il (M.A.B.-D.); 2 Ruth and Bruce Rappaport Faculty of Medicine, Technion—Israel Institute of Technology, Haifa 3200003, Israel; 3 Department of Oncology, Ziv Medical Center, Zefad 1310000, Israel; 4 Department of Surgery, Assuta Medical Center, Tel-Aviv 6971028, Israel; 5 Department of Oncology, Assuta Medical Center, Haifa 3296043, Israel; 6 The Joseph Fishman Oncology Center, Rambam Health Care Campus, Haifa 3498838, Israel; 7 Faculty of Health Sciences, Ben-Gurion University of the Negev, Beer-Sheva 8410501, Israel

**Keywords:** breast cancer, partial-breast irradiation, intraoperative radiotherapy

## Abstract

Background: Intraoperative radiotherapy (IORT) offers single-session treatment during breast-conserving surgery (BCS). Outcomes depend heavily on patient selection and tumor characteristics. Objectives: To assess local recurrence (LR) and prognosis using the 2024 American Society for Radiation Oncology (ASTRO) risk classification in IORT-treated patients. Methods: This multicenter retrospective study analyzed 358 IORT cases (356 patients) treated between 2014 and 2018 using the Zeiss INTRABEAM system. Cases were classified per the 2024 ASTRO partial-breast irradiation guidelines. The primary endpoint was local recurrence-free survival (LRFS); secondary endpoints included overall survival (OS) and mastectomy-free survival (MFS). Findings: The median age was 66 years (range 48–80); all tumors were invasive with a median tumor size of 10 mm. At a median follow-up of 7.1 years, LR occurred in 14/358 cases (3.9%) at a median of 5.2 years post-diagnosis. Five- and 8-year LRFS were 98.3% and 94.8%, respectively; 5- and 8-year OS were 99.4% and 97.7%; MFS at 8 years was 98.2%. Cases that were classified as “conditionally recommended” or “conditionally not recommended” had significantly higher LR than the “suitable” group (8.5% vs. 2.7%; HR 3.25, 95% CI 1.05–10.08, *p* = 0.041). Exploratory analysis showed that cases with ≥2 conditional criteria carried a markedly higher risk than those with 0–1 (21.4% vs. 3.2%; Firth-penalized Cox HR 8.26, 95% CI 2.06–26.06, *p* = 0.005). Conclusions: In appropriately selected patients, IORT achieves local control consistent with contemporary series. The 2024 ASTRO risk classification effectively identifies high-risk cases, supporting its use for risk-adapted candidate selection.

## Simple Summary

Intraoperative radiotherapy (IORT) allows radiation treatment to be delivered during breast-conserving surgery, offering a convenient single-session alternative to conventional postoperative radiotherapy for highly selected patients with early-stage breast cancer. Therefore, appropriate patient selection is critical to achieving optimal outcomes. There is a low level of evidence regarding some features to make this personalized decision. In this multicenter retrospective study, we evaluated long-term clinical outcomes of patients treated with IORT and applied the 2024 American Society for Radiation Oncology (ASTRO) risk classification to assess its ability to stratify recurrence risk. Overall local recurrence rates were significantly low among patients classified as suitable versus patients categorized as conditionally recommended or conditionally not recommended, who experienced higher recurrence rates. These findings support the clinical utility of the updated ASTRO risk classification and highlight its value in guiding risk-adapted patient selection for IORT in routine clinical practice.

## 1. Introduction

Breast cancer is the most frequently diagnosed cancer and a leading cause of cancer mortality in women worldwide. Recent epidemiological statistics highlight its magnitude. According to the Global Burden of Disease (GBD) 2023 Study, the global age-standardized incidence rate of breast cancer in 2023 was 25.5 per 100,000 women (95% UI 22.4–29.0), underscoring its significant public health impact [1]. Due to limited established modifiable risk factors, breast cancer control focuses on early diagnosis and comprehensive management [2]. For early-stage disease, breast-conserving surgery (BCS) followed by adjuvant whole-breast irradiation (WBI) has been the standard of care for decades, supported by randomized controlled trials (RCTs) confirming equivalent oncologic outcomes and long-term safety compared to mastectomy [3,4].

However, conventional WBI requires daily treatment for several weeks, creating a logistical burden and potential radiation-related toxicity [5]. These factors have driven de-escalation strategies, including partial-breast irradiation (PBI) [6]. Among PBI modalities, intraoperative radiotherapy (IORT) delivers a single, targeted radiation dose during lumpectomy, completing surgery and radiotherapy in one session [5,6,7].

RCTs comparing IORT with WBI show mixed results. In the multicenter TARGIT-A trial [8], immediate 50-kV X-ray (photon) IORT met the prespecified 2.5% absolute non-inferiority margin of local recurrence (LR) at the 5-year complete follow-up: LR 2.11% (24/1140) vs. 0.95% (11/1158) with WBI; absolute difference 1.16% (90% CI 0.32–1.99). At a median 8.6-year follow-up, there was no significant difference in local recurrence-free survival (LRFS), HR 1.13 (95% CI 0.91–1.41, *p* = 0.28). By contrast, the single-center ELIOT trial [9] of electron IORT reported higher LR than WBI (11% vs. 2%; HR 4.62, 95% CI 2.68–7.95, *p* < 0.0001). These divergent results underscore the importance of technique selection and patient classification.

The American Society for Radiation Oncology (ASTRO) 2024 guidelines [10] classify PBI candidates using a four-tier system based on the risk for recurrence: suitable, conditionally recommended (CR), and conditionally not recommended (CNR). Both CR and CNR features are defined mostly due to understudied features and experts’ opinions (in earlier ASTRO guidelines, CR and CNR were unified as “Cautionary”). The fourth group is Unsuitable. Recent studies suggest significant differences in outcomes between risk groups [11,12,13].

We conducted a retrospective cohort study of IORT-treated patients at our institution to assess LR and prognosis using ASTRO risk classification, contributing real-world evidence to current guideline debates.

## 2. Materials and Methods

### 2.1. Study Design and Patient Selection

This multicenter, retrospective cohort study was conducted at Assuta Medical Centers (Tel Aviv and Haifa, Israel), and approved by the institutional Helsinki Ethics Committee. We analyzed data of patients diagnosed with early-stage breast cancer who underwent BCS with IORT between September 2014 and December 2018. Eligibility was determined according to ASTRO guidelines [5,14], which evolved over the study period as updates were issued. All cases were reviewed and approved by a multidisciplinary team (radiology, medical oncology, radiation oncology and breast surgery) prior to surgery and IORT. Patients provided informed consent after receiving comprehensive counseling regarding the procedure and potential need for adjuvant WBI. Exclusion criteria included patients with less than 6 months of follow-up and male patients to maintain a uniform cohort consistent with established methodology. The final analytic dataset comprised 356 patients. Two patients underwent bilateral IORT during the same operative session; consistent with Surveillance, Epidemiology, and End Results (SEERs) Solid Tumor Rules (Rule M7) and supported by the contemporary literature [15,16,17,18], bilateral tumors were treated as separate cases, resulting in 358 procedures in 356 patients.

### 2.2. IORT Technique and Clinical Management

IORT was delivered using the Zeiss INTRABEAM system (Carl Zeiss Meditec AG, Jena, Germany) [8], which incorporates a 50 kV X-ray emitting source positioned within spherical applicators ranging from 1.5 to 5.0 cm in diameter, selected during surgery based on surgical cavity dimensions. Following tumor excision and confirmation of negative sentinel lymph nodes (LNs) on frozen section analysis, the appropriate applicator was positioned within the tumor bed. All patients received a prescribed dose of 20 Gy to the applicator surface, with treatment duration ranging from 20 to 50 min depending on applicator size.

Re-excision surgery, adjuvant WBI, systemic chemotherapy, HER2-targeted agents, and endocrine treatment were administered as clinically indicated by the multidisciplinary team according to evidence-based institutional protocols.

### 2.3. Data Collection and Contemporary Risk Classification

Patient data were retrospectively extracted from electronic medical records to a designated institutional database maintained for breast cancer patients undergoing IORT.

Oncologic outcomes were defined according to established practice [19,20], as follows: LR was defined as histologically confirmed invasive carcinoma or carcinoma in situ within the ipsilateral breast; regional recurrence included involvement of ipsilateral axillary, internal mammary, supraclavicular, or infraclavicular lymph nodes; distant recurrence was defined as metastatic disease to distant organs. Contralateral breast cancer was considered a new primary tumor.

Risk classification followed the 2024 ASTRO guideline [10], classifying patients as Suitable, CR, CNR, or Unsuitable. Missing data resulted in designation as Unclassified. Because the ASTRO 2024 guideline [10] characterizes conditional features as based on limited/low-certainty evidence and expert opinion, without clear randomized data separating CR from CNR, and event counts were limited, we pooled CR + CNR into a single conditional category for the primary analysis; this also aligns with earlier ASTRO APBI statements (2009/2017) [5,14], which grouped these features as “Cautionary”. We also conducted an exploratory post hoc analysis comparing high-risk (≥2 conditional criteria) vs. low-risk (0–1), motivated by ASTRO’s statement that PBI may not be appropriate when multiple conditional factors are present. ASTRO-based analyses excluded Unsuitable and Unclassified patients.

### 2.4. Safety and Complications Assessment

Treatment-related complications were categorized as infection, seroma/hematoma, pain/sensory symptoms, skin toxicity, wound complications, and cosmetic changes. Events were considered early if they occurred ≤3 weeks after IORT (aligned with the first oncologic follow-up) and late if >3 weeks.

### 2.5. Statistical Analysis

Median follow-up was calculated using the reverse Kaplan–Meier method to account for censoring patterns. Time-to-event variables were calculated from the date of surgery to the first occurrence of LR, mastectomy, or death, and expressed in years. The primary endpoint was LRFS; secondary endpoints were overall survival (OS) and mastectomy-free survival (MFS). For LRFS, patients were censored at non-local recurrence or death without LR; for OS, death from any cause was the event; and for MFS, mastectomy was the event with censoring at death without mastectomy. Survival functions were estimated using the Kaplan–Meier (KM) method, compared with the log-rank test, and hazard ratios (HRs) with 95% confidence intervals (CIs) were obtained from univariable Cox proportional hazards (PH) regression. Assumption testing used Schoenfeld residuals for PH (*p* > 0.05 acceptable) and dfbetas (threshold|dfbetas| > 1) to screen for influential observations. For risk analyses with limited event numbers, Firth-penalized Cox regression was applied to provide bias-reduced HR estimates accounting for small-sample bias [21].

Categorical associations were tested using Fisher’s exact test due to small expected cell counts. Continuous variables were compared using Mann–Whitney U test after assessment of normality using Shapiro–Wilk tests.

Multivariable Cox proportional hazards regression employed conservative variable selection to ensure model stability. Variables were selected based on clinical relevance and univariable analysis results. Model assumptions were validated using Schoenfeld residuals for proportional hazards testing (*p* > 0.05 considered acceptable), and model performance was assessed using Harrell’s C-index.

All analyses were performed using R version 4.4.3 (R Foundation for Statistical Computing, Vienna, Austria) [22]. All tests were two-sided, with α = 0.05 (*p* < 0.05) considered statistically significant.

## 3. Results

### 3.1. Patient Characteristics and Treatment Outcomes

Between September 2014 and December 2018, 356 women with early-stage breast cancer underwent BCS with IORT; two patients had bilateral treatment in a single operation, yielding 358 treated breasts in total. Patient and tumor characteristics are summarized in Table 1. The median patient age was 66 years (IQR: 60.2–69 years), with 195 patients (54.4%) aged 60–69 years. Thirty-six patients (10.1%) had a prior history of breast cancer: 18 (5.0%) ipsilateral, 17 (4.7%) contralateral, and one (0.3%) unknown laterality. Pre-operative MRI was performed in 154 patients (43.0%). The majority of patients (338/352, 96.0%) underwent lumpectomy with sentinel LN biopsy, with 14 patients (4.0%) undergoing axillary LN dissection.

**Figure 1:**
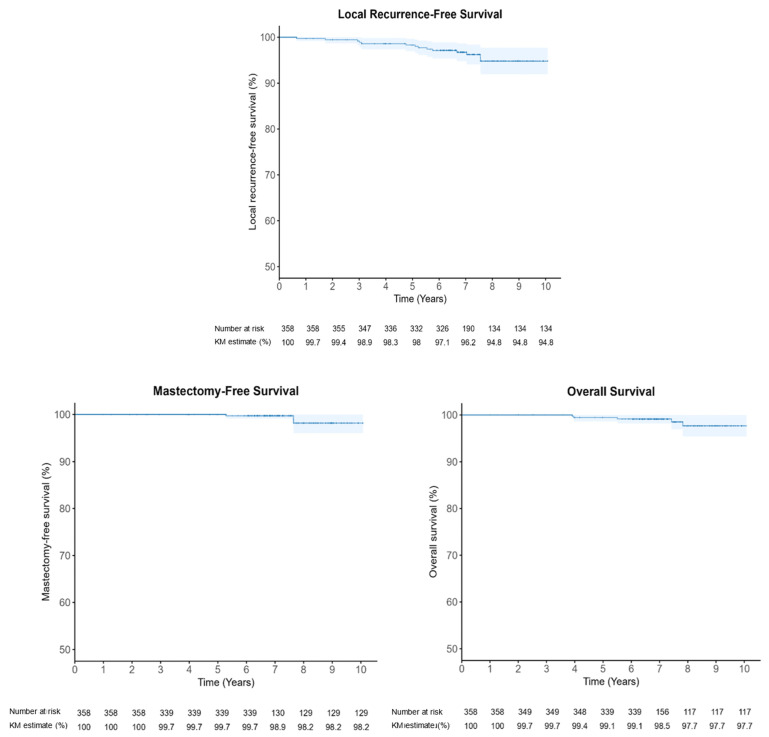
Kaplan–Meier curves for local recurrence-free survival, mastectomy-free survival, and overall survival in the study cohort (*n* = 358). Shaded areas indicate 95% confidence intervals. Numbers at risk and Kaplan–Meier survival estimates (%) are shown below the x-axis.

At a median follow-up of 7.1 years (95% CI: 7.0–7.3), the primary endpoint analysis identified 14 LRs (3.9%), with a median time to LR of 5.2 years (range 0.7–7.6); one patient had locoregional recurrence. As shown in Figure 1, the 5- and 8-year KM estimates of LRFS were 98.3% (95% CI, 96.9–99.7) and 94.8% (95% CI, 91.9–97.7), respectively. For secondary endpoints, 5- and 8-year OS were 99.4% (95% CI, 98.6–100) and 97.7% (95% CI, 95.4–99.9); MFS was 100% at 5 years and 98.2% (95% CI, 96.0–100) at 8 years. Five deaths (1.4%) occurred during follow-up: one due to glioblastoma multiforme (8 years), one age-related (4 years), two non-radiation-related sarcomas (4 and 5.5 years), and one breast-cancer-related death due to metastatic disease (7.5 years). Three patients underwent mastectomy: two after LR and one bilateral mastectomy following recurrence, by patient choice.

Four patients (1.1%) experienced non-local recurrence only (range 4.0–8.8 years): one regional only, two distant, and one combined regional and distant. Overall, 18 patients (5.0%) experienced any recurrence.

All index lesions were invasive carcinoma; 155/358 (43.3%) had an associated DCIS component. Median tumor size was 10 mm (IQR: 8–15 mm), and 186 patients (52.8%) measured ≤10 mm. Histologic grade distribution showed 90 patients (25.6%) with Grade 1; the estrogen receptor was positive in 355 cases (99.2%), and the progesterone receptor was positive in 290 cases (81.0%). HER2 positivity was observed in 14 cases (4.0%). LN involvement was present in 34 cases (9.6%).

### 3.2. ASTRO 2024 Risk Classification and Recurrence Outcomes

**Figure 2:**
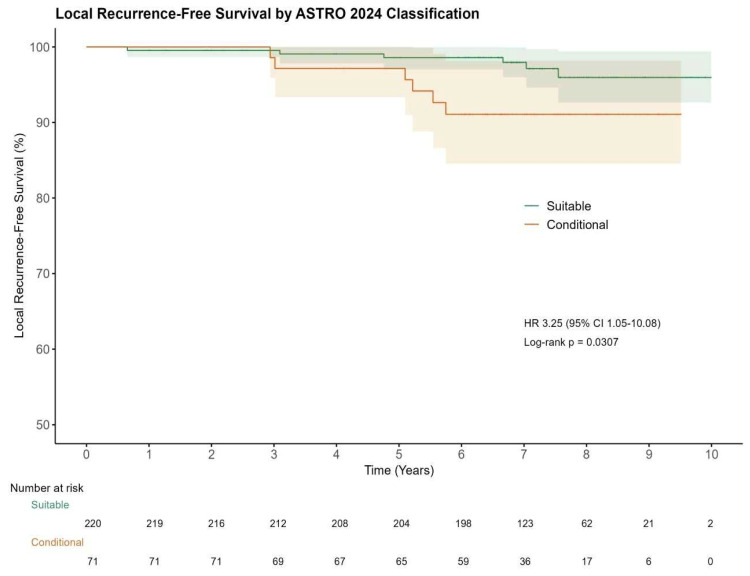
KM curves for LRFS in ASTRO 2024 suitable vs. Conditional (CR + CNR) groups. Shaded bands indicate 95% Cis. Numbers at risk.

Among classified patients (*n* = 341), 220 (64.5%) were Suitable, 71 (20.8%) Conditional (CR + CNR), and 50 (14.7%) Unsuitable (*n* = 17 unclassified due to missing data). LR differed by risk category as shown in Figure 2: 6/220 (2.7%) in Suitable vs. 6/71 (8.5%) in Conditional (4/49 (8.2%) in CR; 2/22 (9.1%) in CNR). Cox regression showed higher hazard for Conditional vs. Suitable, HR 3.25 (95% CI 1.05–10.08, *p* = 0.041); log-rank *p* = 0.0307. PH assumption not violated (*p* = 0.503); all |dfbeta| < 1.

The post hoc risk analysis classified patients into low-risk (*n* = 277) and high-risk (*n* = 14) groups. LR rates were 9/277 (3.2%) vs. 3/14 (21.4%). Cox regression (high- vs. low- risk) yielded HR 7.47 (95% CI 2.02–27.65, *p* = 0.003); log-rank *p* = 0.0004. Given the small event count in the high-risk group (3/14), Firth-penalized Cox confirmed this finding with HR 8.26 (95% CI 2.06–26.06, *p* = 0.005). The PH assumption was not violated (*p* = 0.17); all |dfbeta| < 1.

Several unplanned subgroups demonstrated low event rates: HER2-positive cases (0/14) and node-positive cases (1/34, 2.9%). No LRs occurred among treated breasts that received additional WBI (0/29).

### 3.3. Uni- and Multivariable Histopathologic Analysis

No individual histopathologic characteristic was significantly associated with LR. The highest HRs were observed for grade 3 vs. 1–2, HR 2.46 (95% CI 0.77–7.84, *p* = 0.128); and for involved surgical margins, HR 2.05 (95% CI 0.27–15.67, *p* = 0.489).

Given 14 events (seven events per variable for a two-covariate model), we fit an exploratory multivariable model including the highest HR variables; neither covariate reached significance, with grade 3 vs. 1–2 HR 2.40 (95% CI 0.75–7.66, *p* = 0.140); involved margins HR 1.88 (95% CI 0.25–14.45, *p* = 0.542). C-index 0.617. PH assumption not violated (*p* = 0.43). Complete results of the univariate and multivariable Cox proportional hazards models for LRFS are provided in Table 2.

### 3.4. Treatment Patterns and Adjuvant Therapy

Re-excision surgery was performed in six cases (1.7%) due to close margins. Additional WBI was used in 29 cases (8.1%)—for involved LN (*n* = 17), positive margins (*n* = 5), and unknown reasons (*n* = 7). Median endocrine therapy duration was 3.0 years (IQR: 0.5–4.5 years); ≥5-year duration was observed in 83/358 cases (23.2%). Treatment duration was not associated with LR across 1-, 4-, and 5-year thresholds (Fisher’s exact tests, all *p* ≥ 0.59; Cox models for LRFS, all *p* ≥ 0.41). Pre-operative MRI was associated with a significantly longer biopsy-to-surgery interval (median 42 vs. 32 days; *p* < 0.001, Mann–Whitney U test).

### 3.5. Complications and Safety Profile

Early complications occurred in 51/358 cases (14.2%), including infections (*n* = 22, 6.1%), seromas requiring drainage (*n* = 11, 3.1%), hematomas (*n* = 8, 2.2%), burns (*n* = 3, 0.8%), allergic reactions (*n* = 3, 0.8%), and pleural effusion (1, 0.3%); multiple early complication types occurred in five patients. Late complications were observed in 95/358 cases (26.5%), with pain/sensitivity being most common (*n* = 71, 19.8%), followed by seroma/hematoma formation (*n* = 17, 4.7% combined), infections (*n* = 13, 3.6%), wound complications (*n* = 11, 3.1%), nipple cosmetic changes (*n* = 7, 2.0%), and skin toxicity (*n* = 6, 1.7%). Multiple late complication types occurred in 31 cases (8.7%).

## 4. Discussion

This multicenter retrospective cohort comprised 358 IORT-treated breasts in 356 women and showed excellent 5-year and 8-year LRFS of 98.3% and 94.8%, corresponding to cumulative LR of 1.7% and 5.2%, respectively. Overall, 14/358 (3.9%) were diagnosed with LR at a median follow-up of 7.1 years. Eight-year OS and MFS were 97.7% and 98.2%, respectively. These outcomes are consistent with TARGIT-A [8]: 5-year LR was 2.11% (24/1140) with IORT vs. 0.95% (11/1158) with WBI; at a median follow-up of 8.6 years (maximum 18.9), the IORT arm had 60/1140 (5.3%) LRs, and there was no statistically significant difference between arms in LRFS (HR 1.13, 95% CI 0.91–1.41, *p* = 0.28), MFS (HR 0.96, 95% CI 0.78–1.19, *p* = 0.74), or OS (HR 0.82, 95% CI 0.63–1.05, *p* = 0.13). Notably, eight of our 14 LRs (57%) occurred after 5 years, paralleling TARGIT-A’s 36 of 60 LRs (60%) beyond 5 years and underscoring the importance of extended surveillance after IORT. Similarly, contemporary real-world data from a 1400-patient IORT-only series [12] reported a 5-year LRFS of 94.0%.

By contrast, the single-center phase 3 ELIOT trial (electron IORT) [9] showed higher LR with IORT than WBI (11% vs. 2% at a median of 12.4 years; HR 4.62, 95% CI 2.80–7.95, *p* < 0.0001), while OS was similar. A comprehensive meta-analysis of 15 randomized trials [20], involving 16,474 patients, likewise found that IORT had the highest recurrence risk among PBI modalities compared with WBI (RR 2.79, 95% CI 2.08–3.73, *p* < 0.00001). These divergent results highlight technique dependence and the critical importance of careful patient selection, recognizing that photon- and electron-based approaches differ substantially and may be regarded as separate treatment modalities [14,20,23].

We acknowledge that, as a retrospective single-arm cohort without a contemporary control group, our analyses are not intended to supersede randomized trial evidence or to provide comparative effectiveness versus WBI. Instead, our findings are intended to complement existing trial data by reporting long-term real-world outcomes after IORT as described in other cohorts, registries, and systematic reviews [24,25,26,27,28,29,30]. Specifically, we aimed to assess whether LR patterns observed in clinical practice are consistent with the current ASTRO risk stratification in a hypothesis-generating manner for future prospective validation.

Using the ASTRO 2024 classification [10], LR was higher in Conditional vs. Suitable cases (8.5% vs. 2.7%; HR 3.25, 95% CI 1.05–10.08, *p* = 0.041). In our exploratory post hoc analysis, motivated by ASTRO’s statement that PBI may not be appropriate when multiple conditional factors coexist, accumulating conditional criteria showed a clear gradient: cases with ≥2 criteria had substantially higher LR rates than those with 0–1 (21.4% vs. 3.2%); Firth-penalized Cox confirmed a strong association (HR 8.26, 95% CI 2.06–26.06; *p* = 0.005). These findings are concordant with a recent retrospective comparative study [13] applying ASTRO 2024 categories, which reported similar overall locoregional recurrence with IORT vs. WBI (2.0% vs. 2.3%; *p* = 0.978) at a mean 5.1-year follow-up, but significantly higher locoregional recurrence with IORT within the conditional subgroups CR and CNR: 11.1% vs. 3.0% (*p* = 0.044) and 13.8% vs. 2.5% (*p* = 0.010), respectively. This real-world signal complements ASTRO guidance by emphasizing the cumulative risk burden.

The substantially elevated recurrence risk in cases with multiple conditional criteria (CR/CNR) suggests that this subgroup may represent a higher-risk population within IORT-treated cohorts. These high-risk cases may warrant multidisciplinary discussion of PBI vs. WBI, closer surveillance, and optimization of systemic therapy. Given the 21.4% LR rate among cases with ≥2 conditional criteria, clinicians may consider a low threshold for supplemental WBI in this population. These findings warrant confirmation in larger prospective cohorts and may inform the design of future prospective studies evaluating risk-adapted IORT strategies.

Individual histopathologic factors were not significantly associated with LR in uni- or multivariable analyses, likely reflecting limited power (14 events). Several small subgroups had very low LRs (additional WBI 0/29; HER2-positive 0/14; node-positive 1/34), potentially reflecting effective supplemental treatments in higher-risk cases, though these should be interpreted cautiously due to small numbers. Notably, only half of node-positive cases received supplemental WBI (17/34).

Limitations: Retrospective design; low event counts limiting power; potential referral bias; and evolving selection criteria over time. Although ASTRO guidance became more permissive during the study, previously eligible patients remained eligible, and “unsuitable” criteria changed only modestly; nonetheless, temporal drift may remain. Our pooled CR + CNR analysis assumes similar risk direction across conditional features; although consistent with earlier “Cautionary” grouping and motivated by limited events, results may not apply uniformly to each conditional subgroup. External validation in larger, multi-institutional cohorts is warranted.

Strengths: Consistent institutional protocols, a small team of experienced surgeons under unified oversight, and a dedicated database with mature follow-up provide reliable outcome ascertainment for this specialized technique.

## 5. Conclusions

In this multicenter retrospective cohort of 358 IORT-treated breasts with mature follow-up, IORT was associated with favorable long-term outcomes, demonstrating high 5- and 8-year LRFS alongside high OS and MFS. While LRs were infrequent, notably more than half occurred beyond 5 years, underscoring the need for extended surveillance. Applying the ASTRO 2024 risk classification, cases classified as Conditional experienced a higher risk of LR compared with those classified as Suitable. Furthermore, an exploratory post hoc analysis suggested that the accumulation of multiple conditional risk factors may identify a subgroup with substantially elevated risk. These findings support a risk-adapted approach to patient selection and may inform multidisciplinary decision-making regarding supplemental WBI and surveillance intensity. Given the retrospective design and low event counts, these results should be considered hypothesis-generating and warrant external validation in larger, multi-institutional cohorts and prospective studies.

## Figures and Tables

**Table 1:** Patient and tumor characteristics.

Characteristic	N = 358
Age (years)	66 (60–69)|65 (6)
<60	75/358 (21%)
60-69	195/358 (54%)
≥70	88/358 (25%)
Pre-Surgery MRI	154/358 (43%)
Previous Breast Cancer	36/358 (10%)
Previous Cancer Side	
Contralateral	17/35 (49%)
Ipsilateral	18/35 (51%)
Surgical Procedure	
ALND	14/352 (4%)
SLN	338/352 (96%)
Tumor Side	
Left	193/358 (54%)
Right	165/358 (46%)
Histology	
IDC	164/358 (46%)
IDC + DCIS	155/358 (43%)
ILC	17/358 (5%)
Mucinous	11/358 (3%)
Papillary	6/358 (2%)
Tubular	5/358 (1%)
Grade	
1	90/351 (26%)
2	210/351 (60%)
3	51/351 (15%)
Tumor Size (mm)	10 (8–15) | 11 (5)
≤10 mm	186/352 (53%)
11–20 mm	149/352 (42%)
>20 mm	17/352 (5%)
Margins ‡	
Free	339/353 (96%)
Involved	14/353 (4%)
ER status	
Negative	3/358 (1%)
Positive	355/358 (99%)
PR status	
Negative	68/358 (19%)
Positive	290/358 (81%)
HER2 Status	
Negative	335/349 (96%)
Positive	14/349 (4%)
Ki67	
Ki67 < 14%	222/329 (67%)
Ki67 ≥ 14%	107/329 (33%)
LN Removed	2.0 (1.0–3.0) | 2.3 (1.6)
LN Involved	
0	321/355 (90%)
1	29/355 (8%)
2	3/355 (1%)
3	1/355 (0.3%)
8	1/355 (0.3%)
ASTRO 2024 Classification¶	
Suitable	220/341 (65%)
CR	49/341 (14%)
CNR	22/341 (6%)
Unsuitable	50/341 (15%)

‡ Margins were defined as “involved” if tumor was present at the inked surface (“tumor on ink”) and “free” otherwise. ¶ CR = conditionally recommended; CNR = conditionally not recommended. Continuous variables: Median (Q1–Q3) | Mean (SD); Categorical variables: n/N (%). Percentages calculated using available data for each variable as denominator. Missing values are excluded from calculations and not displayed.

**Table 2:** Univariable and multivariable Cox proportional hazards models for local recurrence-free survival.

Predictor	Comparison	Univariable Cox		Multivariable Cox (Exploratory)	
		HR (95% CI)	*p*-Value	HR (95% CI)	*p*-Value
ASTRO 2024 risk group	Conditional vs. Suitable (reference group)	3.25 (1.05–10.08)	0.041	-	-
Post hoc risk group	High-risk vs. Low-risk (reference group)	7.47 (2.02–27.65)	0.003	-	-
Post hoc risk group	High-risk vs. Low-risk (reference group)	8.26 (2.06–26.06) *	0.005	-	-
Tumor grade	Grade 3 vs. Grade 1–2 (reference group)	2.46 (0.77–7.84)	0.128	2.40 (0.75–7.66)	0.14
Surgical margins	Involved vs. Not involved (reference group)	2.05 (0.27–15.67)	0.489	1.88 (0.25–14.45)	0.542
Endocrine therapy duration	≥1 year vs. <1 year (reference group)	0.99 (0.31–3.13)	0.981	-	-
Endocrine therapy duration	≥4 years vs. <4 years (reference group)	0.64 (0.22–1.85)	0.41	-	-
Endocrine therapy duration	≥5 years vs. <5 years (reference group)	0.78 (0.23–2.64)	0.691	-	-

* Univariable Firth-penalized Cox regression.

## Data Availability

The data presented in this study are available on request from the corresponding author. The data are not publicly available due to ethical and privacy restrictions.
